# Trade-offs between growth, reproduction and defense in response to resource availability manipulations

**DOI:** 10.1371/journal.pone.0201873

**Published:** 2018-08-22

**Authors:** Juliana Tuller, Robert J. Marquis, Samara M. M. Andrade, Angelo B. Monteiro, Lucas D. B. Faria

**Affiliations:** 1 Setor de Ecologia e Conservação, Departamento de Biologia, Universidade Federal de Lavras, Lavras, MG, Brazil; 2 Department of Biology and the Whitney R. Harris World Ecology Center, University of Missouri-St. Louis, St. Louis, MO, United States of America; Banaras Hindu University, INDIA

## Abstract

The Brazilian Cerrado is one of the most endangered biomes in the world. We evaluated the sustainability of leaf harvest in one of the most important Cerrado tree species, *Stryphnodendron adstringens*. The bark of this tree is used as a source of medicinal tannin. Harvesting bark, however, often kills the tree. In a manipulative field experiment, we tested the hypothesis that harvesting leaves, which might serve as an alternative source of tannin, would be less detrimental for tree survival, growth, reproduction, and defense than harvesting bark. In a two-way crossed experimental design, we either clipped 100% of a plant’s leaves or applied NPK fertilizer to the soil. Our predictions of the experimental outcomes were based on plant resource and defense theory. Growth was determined by total leaf dry mass production, reproduction by inflorescence and fruit production traits, and defense by total phenolics, hydrolyzable tannins, and condensed tannins. Fertilization had a marginally positive effect on total leaf dry mass. Defoliation had no effect on subsequent leaf production, and most importantly, no plants died as a result of defoliation. We found high tannin amounts in leaves of *S*. *adstringens* produced both prior to and subsequent to clipping, further suggesting that leaves could serve as a sustainable alternative source of tannin. After clipping, plants invested more in tannin production and less in reproduction. Our results suggest that leaf harvest may be more sustainable than harvesting of bark in *S*. *adstringens*. We suggest the need for further investigation of the medicinal properties of leaf tannins to formulate a viable sustainable management plan for the exploitation of this plant species.

## Introduction

Plants allocate resources to maximize their fitness in the face of varying abiotic (e.g., nutrient availability) and biotic (e.g., herbivory) limitations. Limited resources may result in conflicting demands for such resources, with the result that plants may not be able to invest simultaneously in growth, reproduction and defense [1, 2, 3]. According to the ‘defense trade-off hypothesis’, plants may respond to resource limitations by making 'choices', that is, by investing in one or two functions instead of all, or investing more in one kind of anti-herbivore defense than another [4, 5, 6].

Herbivory is an interaction between plants and their consumers with important ecological and evolutionary consequences [7, 8]. The amount of plant tissue lost to herbivores is often determined by biotic and abiotic conditions that affect the quality and quantity of plant defense [9]. In turn, biotic and abiotic conditions also influence plant responses to herbivory [10]. The ‘continuum of plant response to herbivory hypothesis’ predicts that the impact of herbivory may be detrimental, neutral, or beneficial for plant fitness depending on the plant's ability to replace tissue consumed by herbivores as influenced by the abiotic environment [10, 11]. For example, plants may delay fruit production or produce fewer fruits following herbivore attack [12, 13]. However, as a counterbalance, fertilization might positively affect the production of flowers, fruits and seeds [14].

Plants have evolved several strategies to avoid herbivore attack, such as associations with ants, physical barriers, and production of secondary chemical compounds [15, 16]. Phenolics are secondary chemical compounds commonly produced by plants, and often are involved in defense against herbivorous insects and pathogens [17, 18]. In high concentrations, phenolics can injure herbivore guts [19]. Generally, they have a negative effect on insect fitness [20].

Tannins are a class of phenolic compounds most commonly produced by plants [19], coming in either hydrolyzable or condensed form [21]. They function as defensive compounds by precipitating proteins, causing oxidative stress, and by disrupting membranes [22]. The impact of increasing tannin concentration varies greatly, sometimes negatively affecting insect fitness, and sometimes positively so, depending on the insect species [19]. Tannin production varies with the plant's genotype, development stage, and environmental conditions (e.g., stress in response to nutrient availability or injuries) [19, 23]. In addition, the production of tannins and other defensive compounds may vary among plant organs [24]. The ‘optimal defense hypothesis’ suggests that the highest defended plant organs are those that represent the highest fitness value, considering energetic costs and/or vulnerability to herbivory [24, 25, 26].

*Stryphnodendron adstringens* (Mart.) Coville (Fabaceae: Mimosoideae), known locally as barbatimão, is a common tree species of the Cerrado biome (Brazilian Savanna). This species produces racemose andromonoecious inflorescences that are visited by bees, wasps, flies, butterflies, and beetles, the first two groups representing more than 90% of all visits [27]. *S*. *adstringens* fruit production is low relative to the number of flowers produced and can be resource limited [27]. Inflorescences are produced from July to November and fruit production occurs throughout the year [28]. Moreover, senescent and resprouting leaves occur at the same time as fruit ripening, from July to November [28].

*Stryphnodendron adstringens* has high tannin levels in its tissues, 25%-35% of dry mass [29, 30]. Tannins represent an important source of antioxidants in human diet [18, 21]. For this reason, barbatimão is an important medicinal plant for rural populations in Brazil. There are several pharmacological studies of the use of this species as a medicine against numerous diseases [31, 32]. Rural populations sometimes overexploit the bark of *S*. *adstringens* as a source of tannin, often removing more than 50% of the bark, which can result in tree death [33, 34]. Some studies have suggested sustainable management strategies for this species, supporting the active management of populations of *S*. *adstringens* [33, 35]. Some authors have proposed harvest plans that would allow sufficient harvest to improve medicine production but at the same time conserve the species [36, 37]. However, there are no experimental studies that have deliberately manipulated tissue harvest levels of *S*. *adstringens* to determine the impact of that harvest on plant growth and survivorship, reproduction, and defense.

The Cerrado biome is characterized by low soil fertility [38] and relatively high levels of herbivory [39, 40]. Fertilization might be used to mitigate the effects of leaf harvest [10, 11]. Thus, a sustainable harvest plan could link fertilization with leaf harvest.

In this study, we evaluated the impact of leaf harvest in the context of plant allocation and defense theory. Considering a gradient from lowest to highest resource availability across four treatments (low to high: Clipped (C), Clipped-Fertilized (CF), Control (Co), and Fertilized (F)), the following hypotheses were tested: i) the 'trade-off hypothesis': all plants will invest in leaf production over reproduction to maintain photosynthetic activity. Furthermore, higher resource availability will increase investment in reproduction, while artificial damage will increase defense investment, suggesting a trade-off between reproduction and defense; ii) the 'continuum of plant response to herbivory hypothesis': resource availability will determine plant investment in reproduction and defense, CF plants are less resource limited than C plants, resulting in the lowest leaf replacement by C plants, but a neutral or positive impact on CF plants; and iii) the 'optimal defense hypothesis': the highest defended tissue will be the leaves, the most important tissue to produce photosynthetic metabolites. Overall, we hypothesized that harvesting leaves of *S*. *adstringens* as a source of medicinal tannin would negatively affect plant growth but not as much as the harvest of bark, and that fertilization would ameliorate harvest effects.

## Materials and methods

### Study area

The study sites were in the Itumirim municipality, in the state of Minas Gerais, Brazil (Site 1: 21°14'9.82"S 44°49'43.29"W; Site 2: 21°13'41.98"S, 44°51'0.43"W). This region is characterized by mild summers and dry winters, with an annual average temperature of 19.4°C, and an annual average rainfall of 1,530 mm [41]. The original vegetation of this region was seasonal forest of the Atlantic Forest biome [42] in transition with the Cerrado biome. Most of the native forest is very fragmented by human activity, such as coffee crops, pastures and human settlements.

### Sampling design and field experiment set up

In a crossed experimental design, we either completely defoliated (clipping leaves) plants or not, crossing this treatment with the addition of fertilizer to the soil of experimental plants or not. We tested the impacts of these treatments on tannin production by different plants organs, and on plant survivorship, growth, reproduction, and defense against future attack.

In April 2014, we selected two sites, 2.4 km apart, marking 40 reproductive individuals of *S*. *adstringens* at each. Plant nutrient availability was either increased by adding fertilizer and/or decreased by clipping all leaves using a tree pruner. At each site, ten individuals were randomly assigned to one of the following treatments: (i) control (Co); (ii) application of 600 g of NPK fertilizer to the soil (F); (iii) 100% leaf removal by clipping (C); (iv) application of 600 g of NPK fertilizer to the soil and 100% leaf removal by clipping (CF).

In April 2014, all leaves from plants assigned to treatments C and CF were clipped. From April 2014 to February 2015, on alternative months, 100 g of NPK (10-10-10) fertilizer were applied to the soil around plants assigned to the treatments F and CF. The fertilizer was applied to three holes around each plant to avoid loss through surface runoff.

### Plant vegetative growth

We dried and weighed all the leaves clipped from plants assigned to treatments C and CF to estimate the investment in vegetative biomass up until the beginning of the experiments. In May 2014, one month after we clipped those plants, we counted the number of leaves produced by each plant to estimate the immediate response to clipping. At the end of the experiment, in June 2015, we clipped all leaves from all plants using a tree pruner to measure investment in vegetative biomass by each plant. We dried all leaves at 40°C for 72 h, and then weighed them. To summarize, we collected vegetative data on total leaf dry mass of C and CF plants before treatments; the number of leaves produced by C and CF plants one month after they were clipped for the first time; and the total leaf biomass of all plants at the end of the experiments.

### Plant reproductive investment

We measured fruit and seed production, in response to the treatments, because we wished to know whether leaf harvest might affect future population size via a change in seed production. In April 2014, we collected all fruits from all plants before setting up the field experiments. We counted and dried all fruits at 40°C for 72 h and weighed them to determine plant's reproductive investment before the treatments. In June 2015, fourteen months after the field experiment started, we collected all fruits again, counted, and determined the total biomass per plant. In addition, we visited the study area monthly, from May 2014 to June 2015, to count the number of inflorescences. We designated the maximum number of inflorescences observed at the field as the total number of inflorescences produced by each individual subsequent to the initiation of the treatments. Fruit set was calculated as the number of fruits produced per inflorescence. The impact of the treatments on fruit production (henceforth impact on fruits) was estimated as the difference between the number of fruits produced before and after treatments.

In March 2017, we visited all the plants and counted all fruits to check if plants were reproducing normally again.

### Secondary compound analyses of leaves, fruits, and seeds

We randomly collected four leaves from each plant to determine leaf tannin content. For fruit and seed analysis, we opened a maximum of 75 fruits per plant, classifying the seeds as apparently healthy or injured by insects. We x-rayed apparently healthy seeds to verify which seeds contained insect larvae. We dried and milled leaves, undamaged seeds and the fruits to assess their total phenolic, condensed tannin and hydrolyzable tannin concentrations.

We purified tannins from leaves, fruits and seeds using Sephadex LH-20 to produce three bulk extractions, one for each plant organ [43]. We used purified tannin powder from those plant tissues to build one standard curve for each plant organ and chemical analysis. Standard curves were built using known concentrations of purified tannin; their related absorbance values obtained from a spectrophotometer [43]. We only considered r^2^ ≥ 0.95 for standard curves. Finally, we prepared each individual sample extract with an acetone and L-ascorbic acid solution to conduct the three defensive compound analyses. Total phenolic concentration of each sample was estimated using Folin-Denis assay [44], condensed tannins by a 95% butanol: 5%HCl assay [45], and hydrolyzable tannins by potassium iodate assay [46].

### Data analyses

We performed analysis of variance (ANOVA) with Tukey post-hoc tests to evaluate the variation in chemical concentrations as affected by treatments, i.e., hydrolyzable tannins, condensed tannins, and total phenols, among plant tissues, leaves, fruits and seeds. Further, in order to assess trade-offs between chemical compound production and reproduction, we calculated Pearson correlations between different chemical compounds in the same plant tissue, and the same chemical compound between different plant tissues of all plants. The P-values for both correlations were corrected using Holm’s Method to correct for multiple comparisons [47]. Finally, we performed ANOVA with Tukey post-hoc tests to evaluate variation in defense, growth and reproduction between treatments. We also performed ANOVA to compare fruit production of plants from the different treatments and controls in 2017. We assessed defense and reproduction as latent variables, using dimension reduction after a principal component analysis (PCA). Considering the first component, which captured the largest amount of covariance among variables, we computed defense as the covariance between total phenols, hydrolyzable tannins, and condensed tannins, on one hand; and reproduction as the covariance between number of inflorescences, fruit set, and fruit biomass, on the other hand. Further, we used leaf dry mass (g) as an estimate of plant growth. Also, for plants of clipping treatments (C and CF), we used a linear regression to assess the relationship between the leaf dry mass before clipping and number of leaves produced after clipping.

Although differences between treatments can be assessed with multivariate distances between points (e.g., PerMANOVA), using a single dimension approach permits us to use the covariance between variables, and their rotations in relation to the latent variable, to infer more directly about increases or decreases in defense and reproduction between treatments, and directly compare to plant’s growth. However, although the first dimension captures most of the variance, a single dimension may omit important information, which may be present in other dimensions. It was not possible to evaluate fruit and seed chemical compounds among treatments, because most clipped plants did not produce sufficient fruit and seed material. All analyses were conducted using R software [48].

## Results

**Fig 1:**
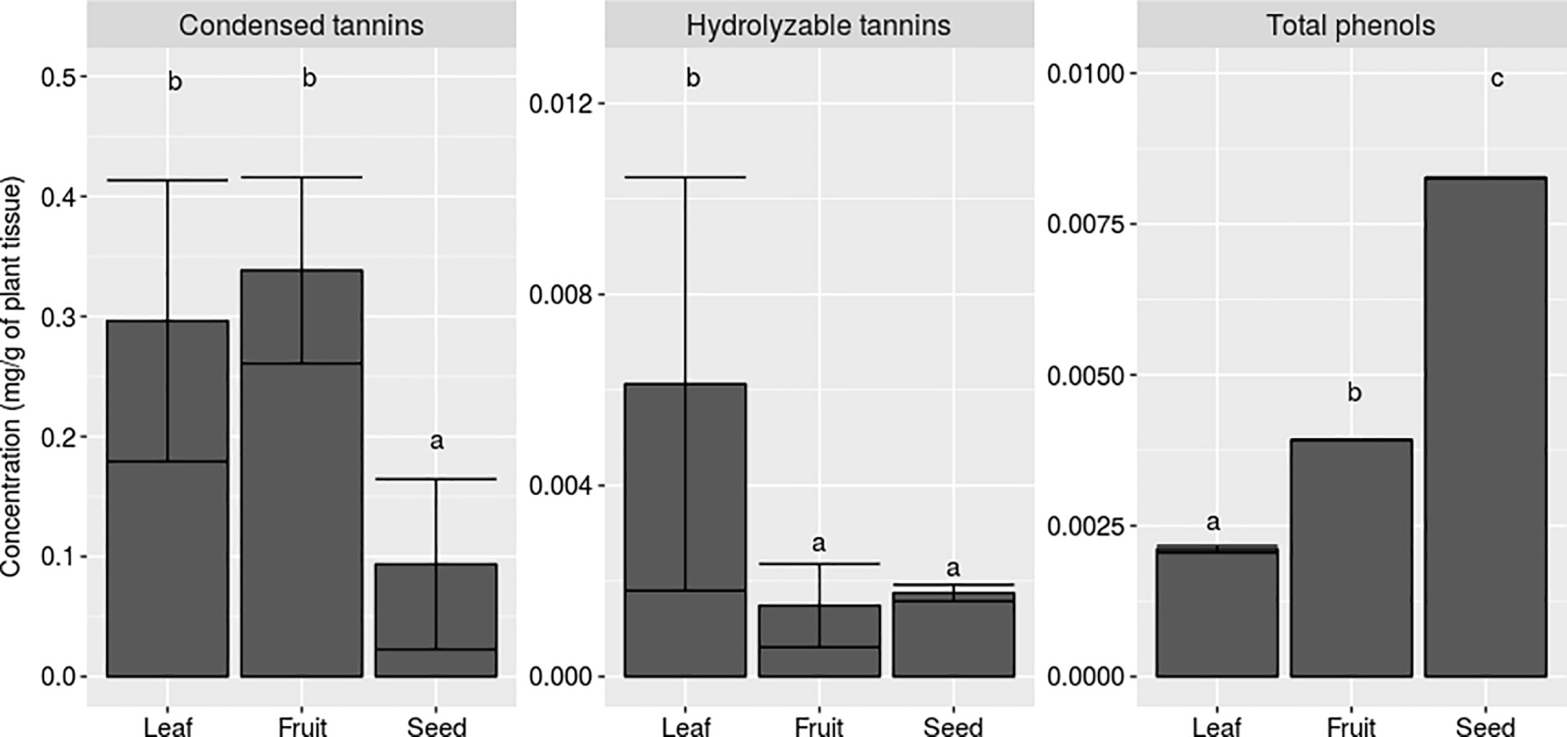
Concentration of chemical compounds in different plant organs. Means and standard deviations for the concentration of (A) total phenols, (B) hydrolyzable tannins and (C) condensed tannins on leaf, fruit and seed dry mass of *Stryphnodendron adstringens*. (p< 0.001, F = 120.43; p< 0.001, F = 27.57; p< 0.001, F = 28.52, respectively). Different letters represent significant differences for a significant level of 0.05. Number of plants observed: 69 for leaves, 38 for fruits and 13 for seeds.

During the experiment, six plants died, which were two CF plants at site 1, and two C, one Co and one F plant at site 2. Secondary compound concentration varied significantly among leaves, fruits and seeds of *S*. *adstringens* (Fig 1). Fruits and leaves had higher condensed tannin concentrations than seeds (p< 0.001, F = 120.043, Fig 1A). Conversely, leaves produced more hydrolyzable tannins than fruits and seeds (p< 0.001, F = 27.57, Fig 1B), while seeds produced more total phenols, followed by fruits and leaves (p< 0.001, F = 28.52, Fig 1C). We also found positive correlations between hydrolyzable tannins in leaves and hydrolyzable tannins in fruits (Table 1), and between condensed tannins and total phenols in leaves (Table 2). However, there was no correlation between chemical secondary compounds and different tissue types of *S*. *adstringens* (S1 Table).

**Table 1:** Correlations between chemical secondary compounds—mg/g of plant tissue dried weight for total phenols, hydrolyzable tannins, and condensed tannins—In plant’s tissues–leaves, fruits and seeds—In *Stryphnodendron adstringens*. P-values were corrected using Holm’s method [48].

Variables		Correlation	P-value	N
**Leaf hydrolyzable tannins**	**Leaf condensed tannins**	**0.33**	0.005*	**43**
**Leaf total phenols**	**Leaf hydrolyzable tannins**	**-0.44**	<0.001*	**48**
**Leaf total phenols**	**Leaf condensed tannins**	**-0.29**	0.017*	**63**
**Fruit hydrolyzable tannins**	**Fruit condensed tannins**	**0.33**	0.046*	**35**
Fruit total phenols	Fruit hydrolyzable tannins	-0.02	0.892	48
Fruit total phenols	Fruit condensed tannins	<0.01	0.991	56
**Seed hydrolyzable tannins**	**Seed condensed tannins**	**-0.77**	0.002*	**49**
Seed total phenols	Seed hydrolyzable tannins	-0.20	0.510	63
Seed total phenols	Seed condensed tannins	0.37	0.218	52

*represents statistically significant results, considering p ≤ 0.05

**Table 2:** Correlations in chemical secondary compounds—mg/g of plant tissue dried weight for total phenols, hydrolyzable tannins, and condensed tannins—Between plant’s tissues–leaves, fruits and seeds—In *Stryphnodendron adstringens*. P-values corrected using Holm’s method [48].

Variables		Correlation	P-value	N
Leaf total phenols	Fruit total phenols	0.09	0.604	68
Leaf total phenols	Seed total phenols	-0.21	0.498	67
Fruit total phenols	Seed total phenols	0.11	0.722	67
Leaf condensed tannins	Seed condensed tannins	0.21	0.497	49
Fruit condensed tannins	Seed condensed tannins	0.11	0.722	46
Leaf condensed tannins	Fruit condensed tannins	-0.16	0.326	55
Leaf hydrolyzable tannins	Seed hydrolyzable tannins	-0.09	0.733	43
Fruit hydrolyzable tannins	Seed hydrolyzable tannins	-0.12	0.698	44
Leaf hydrolyzable tannins	Fruit hydrolyzable tannins	0.23	0.169	47

**Fig 2:**
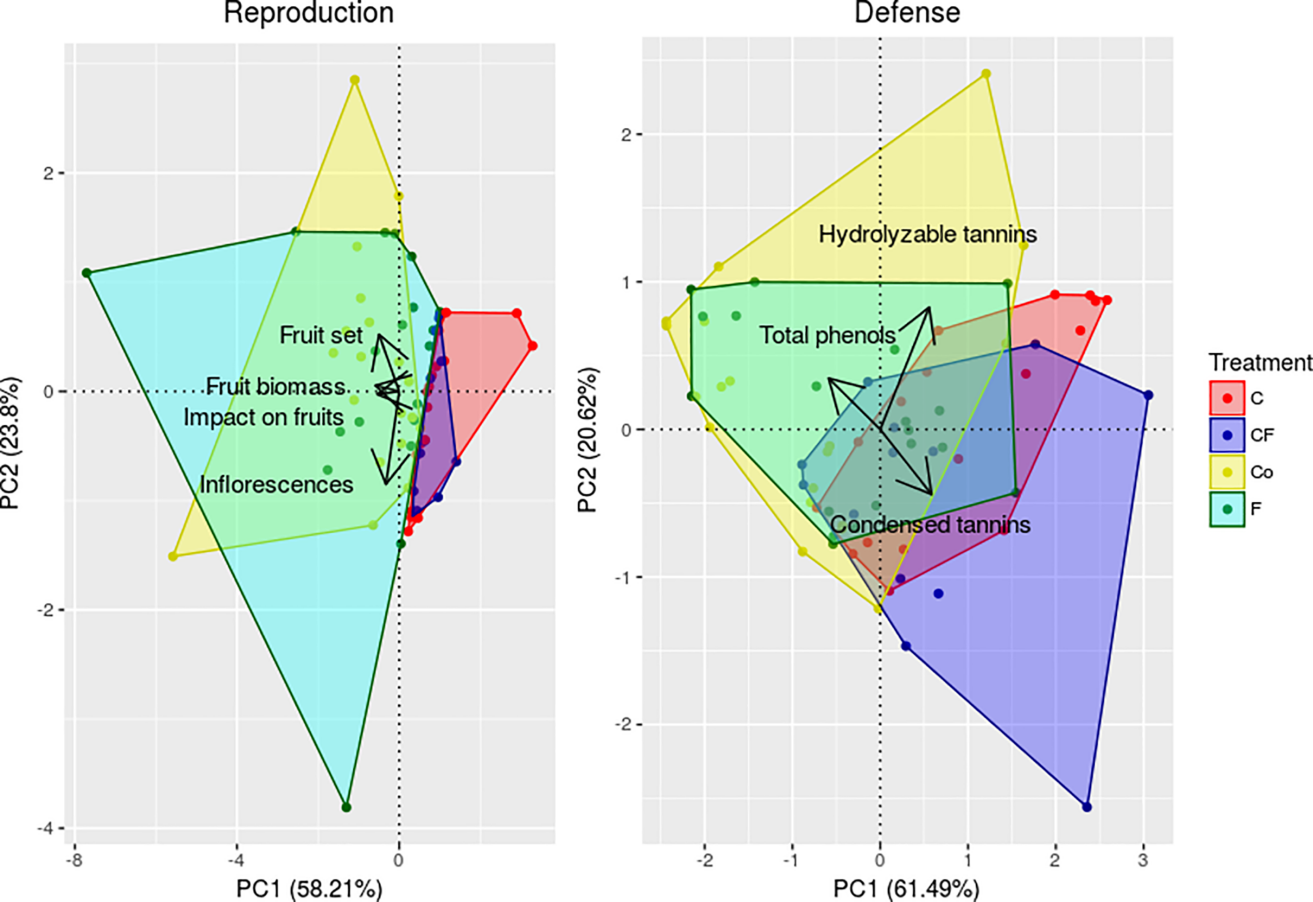
Principal component analysis of reproductive and defensive traits. Principal component analysis for the covariance (A) among reproductive traits: number of inflorescences, fruit set, impact on fruits and fruit biomass (g); and (B) among defensive traits: total phenols, hydrolyzable tannins, and condensed tannins (mg/g of plant tissue). The first dimension (PC1) was used as latent variable for each trait. Number of plants observed: 17 C, 13 CF, 19 F and 19 Co. Plant treatments: C = Clipped; CF = Clipped-Fertilized; F = Fertilized; Co = Control.

The number of inflorescences, fruit set, and number and biomass of fruits were negatively correlated with the first axis (PC1) in the principle component analysis. The variance explained by this latent variable was 58.2%. Thus, an increase in the reproductive PC1 reflects a decrease in reproductive output (Fig 2A). Regarding the dimension reduction for defensive chemical compounds, we found that hydrolyzable and condensed tannin concentrations were positively correlated with PC1, while total phenols had a negative correlation with PC1. Thus, we interpret an increase in the defense PC1 to reflect an increase in overall defense. The variance explained on PC1 was of 61.5% (Fig 2B).

**Fig 3:**
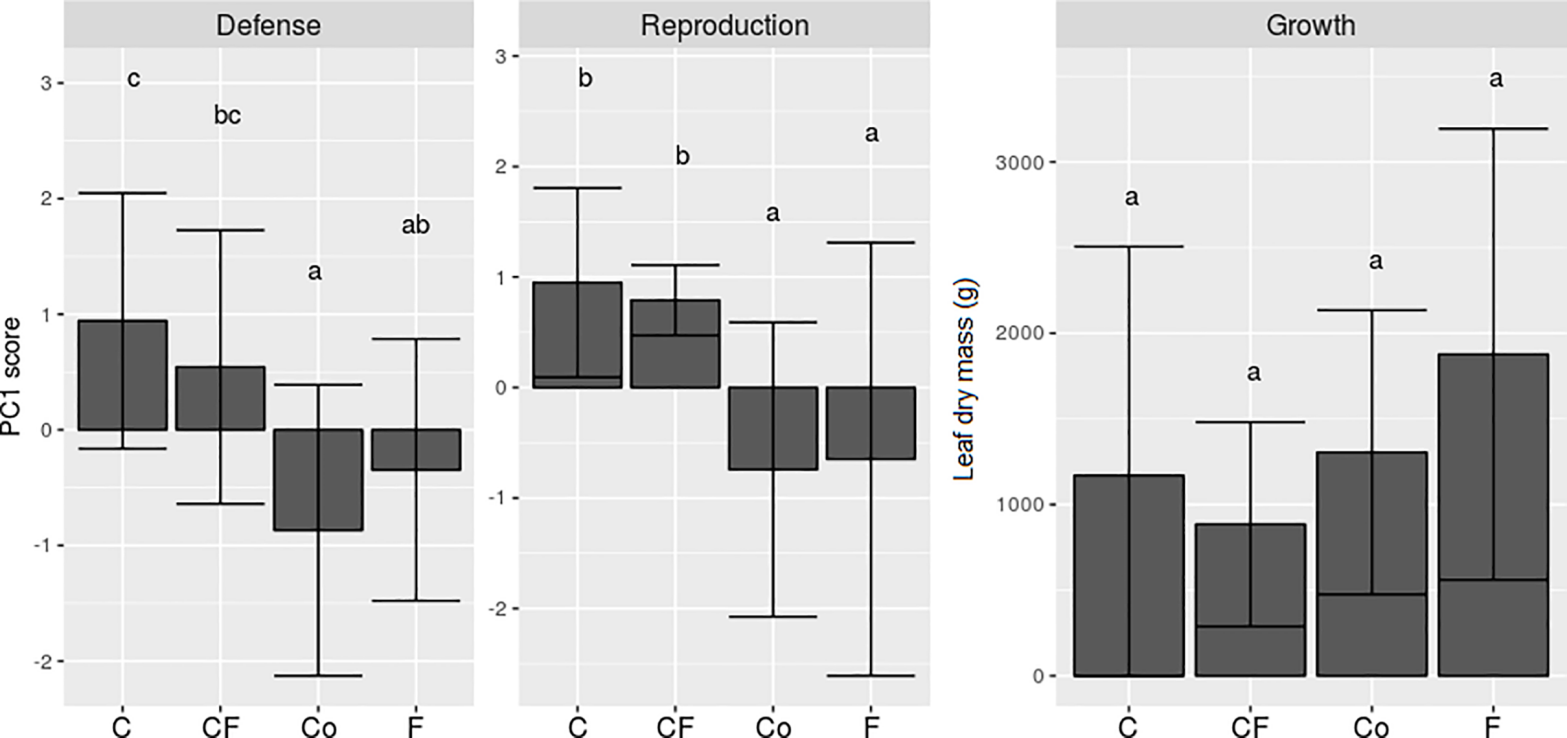
Defense, reproduction and growth investment among plant treatments. Means and standard deviations of (A) defense, (B) reproduction and (C) growth in *Stryphnodendron adstringens* after different treatments: C, CF, Co and F (p< 0.01, F = 8.62; p< 0.001, F = 7.84; p = 0.074, F = 2.42, respectively). Defense and reproduction were evaluated as latent variables after a dimension reduction analysis (PCA). Increases in defense scores translate into higher plant defense, while increases in reproductive scores translate into lower reproductive capacity. Number of plants observed: 17 C, 13 CF, 19 F and 19 Co. Plant treatments: C = Clipped; CF = Clipped-Fertilized; F = Fertilized; Co = Control.

**Fig 4:**
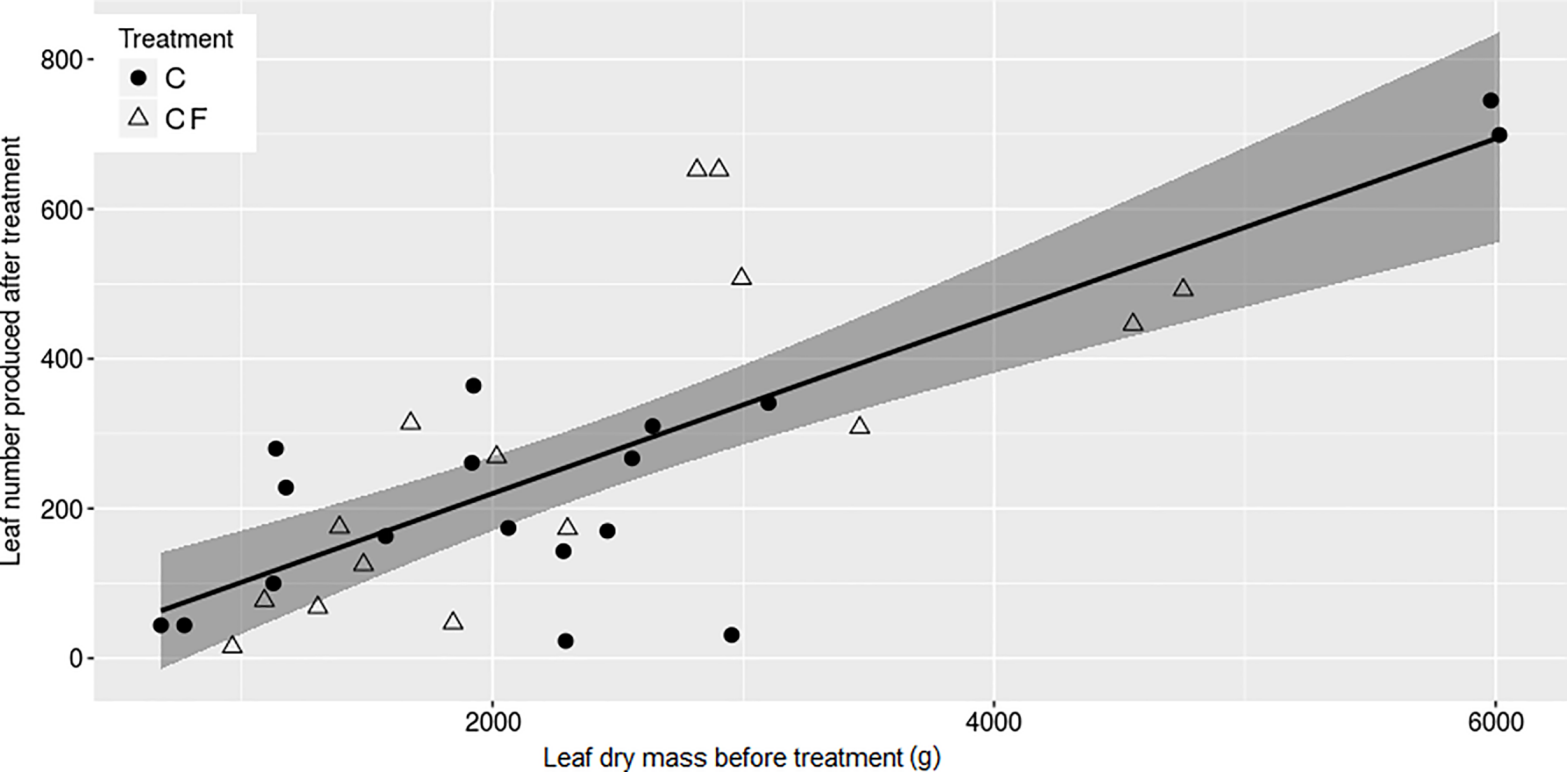
Leaf production before and after treatments. Number of leaves produced by *Stryphnodendron adstringens* of the treatments CF and F one month after clipping treatments as a function of their leaf dry mass before. Linear regression model with 95% confidence intervals in dark gray: p< 0.01, F = 44.71, y = -16.92 + 0.12 * x. Number of plants observed: 18 C and 15 CF. Plant treatments: C = Clipped; CF = Clipped-Fertilized.

Clipping increased allocation to defense, while fertilization had no effect on defense production (p< 0.01, F = 8.62, Fig 3A). In contrast, clipping decreased reproductive output, and again, fertilization had no affect compared to controls (p< 0.001, F = 7.84, Fig 3B). Although fertilized plants had the highest leaf dry mass, we did not find significant differences among treatments in this variable (p = 0.074, F = 2.42, Fig 3C). However, larger plants, i.e., those that had more leaves at the beginning of the experiment, produced greater leaf dry mass after clipping (p< 0.01, F = 44.71, Fig 4). Two years after we finished our experiment, in March 2017, the plants from all treatments were reproducing similarly and produced the same quantity of fruits (p = 0.829, F = 0.29).

## Discussion

This is the first study to quantify the impact of experimental harvest and management on *Stryphnodendron adstringen*s. We hypothesized that leaf harvest, as a means of extracting tannins, would negatively affect tree growth but that fertilization would ameliorate this impact. Contradicting our hypothesis, leaf harvest did not affect leaf production but leaf harvest reduced fruit production. In contrast to the negative effect of bark harvest reported in previous studies [29, 30], survivorship was not decreased by leaf harvest. In addition, the plants recovered their fruit production two years after being clipped. Fertilization had a marginally positive effect on leaf production but only for the non-clipped treatment, and did not impact reproduction. Plants that had high leaf dry mass initially had high leaf production subsequent to defoliation. This result suggests that there is an influence of local environmental conditions and/or plant genotype on growth and reproduction. The clipping treatment induced a trade-off in plant response, such that reproduction decreased but tannin concentration increased in plant tissues. The 'continuum of plant response to herbivory hypothesis' was not corroborated, since plants of both clipped treatments produced similar total leaf dry mass, independent of the fertilization treatment. We observed that leaves, fruits and seeds contain different levels of total phenols, hydrolyzable tannins and condensed tannins. As predicted by the 'optimal defense hypothesis', leaves were the highest defended tissue. In addition, we observed both positive and negative correlations (the latter indicating trade-offs) in the production of secondary compounds in leaves, fruits and seeds.

This species showed a trade-off between reproduction, on the one hand, and growth and defense on the other. Plants invested fewer stored resources in reproduction and more in defense following defoliation. Severe damage requires allocation of resources to replace lost leaf tissue [12, 49]. Maintenance of leaf production in the face of defoliation apparently reduced reproduction in this study. Higher levels of defense in newly produced leaves may have further compounded the tradeoff. Inducible defense is an important strategy, considering that herbivory varies in space and time [6, 50]. Tannins often have a defensive function against herbivores [17, 18, 19]. Our results are consistent with the hypothesis that damage induces higher secondary compound production to reduce the likelihood of subsequent herbivore damage [17]. In addition, investing in defensive compounds following herbivore attack instead of reproduction may be more profitable for long-lived trees, like *S*. *adstringens*, because they may have many future reproductive episodes.

After clipping, we observed that condensed tannin and hydrolyzable tannin concentrations increased, while total phenolics decreased. Thus, levels of condensed and hydrolyzable tannins showed a positive correlation. In contrast, we observed a trade-off between condensed tannins and total phenols, and between hydrolyzable tannin and total phenol production in plant tissues. Producing multiple kinds of defense helps the plant avoid damage by a variety of herbivorous insect species but may result in trade-offs among different kinds of defenses [4], as observed in this study.

Contradicting the 'continuum of plant response to herbivory hypothesis', damaged plants with higher versus lower resource availability (i.e., CF and C plants) did not differ in leaf production following defoliation. Fertilization had only a marginal effect on growth (leaf biomass production), but did not affect reproduction or defense. *S*. *adstringens* has associations with *Rhizobium* and nodule formation [51], and high nutrient availability might inhibit this association [52, 53]. In this way, plants might have similar nutrient availability under fertilized and non-fertilized conditions, resulting in approximately equal investment in growth, reproduction, and defense. It is important to note that this plant species showed a high ability to replace its leaves after severe damage. Whether they could do so following multiple defoliations awaits further experimentation. Maintenance of leaf area in the face of herbivore attack is critical for maintenance of functions [54].

Artificial damage also affected plant defense, as clipped plants reduced total phenol production and increased hydrolyzable and condensed tannin production in plant tissues. Individuals of *S*. *adstringens* differentially invest in the defense of leaves, fruits and seeds. Leaves and fruits have around 30% of their biomass composed of condensed tannins, while seeds have less than 10%. Leaves also have the highest hydrolyzable tannin amount of all tissues, but the lowest total phenol amount; fruits have low hydrolyzable tannins and intermediate total phenol amounts; seeds have a high total phenol amount, but very low quantities of the other two. Leaves should be highly defended tissues due to their great importance for photosynthesis [49]. Therefore, our results are partially consistent with the 'optimal defense hypothesis', because leaves had the highest production of condensed tannins and hydrolyzable tannins, but they did not have more total phenols. Studies about differences in the investment of secondary compounds production help to determine which tissue would be more profitable for commercial exploitation [55]. In the case of *S*. *adstringens*, high leaf tannin concentrations found in leaves suggests a commercial use in place of bark for medicine production.

Currently, popular medicine only uses the bark of *Stryphnodendron adstringens*, causing the death of overexploited plants [29, 30]. We observed that leaves of *S*. *adstringens* have similar tannin concentrations to bark, as reported in the literature [56], and complete defoliation neither kills trees nor reduces their growth. These results together suggest that leaves could replace bark as the exploited resource without the concomitant negative impact on tree survivorship. How plants might respond to multiple defoliations is yet to be determined. Nor do we know the impact of leaf and bark harvest on plant population viability via reduced reproduction. Although there are several studies about the use of *S*. *adstringens* bark for medicinal purposes, only a single study has focused on leaves [57]. Thus, we suggest that pharmacological studies should focus on the use of leaves of *S*. *adstringens* as a source of medicine as this may ultimately lead to a more sustainable exploitation of this plant species.

Authors' contributions statement: JT, RJM and LDBF conceived the ideas and designed the methodology; All the authors collected the data; JT and ABM analyzed the data; JT wrote the manuscript. All authors contributed critically to manuscript drafts and gave final approval for submission for publication.

## Supporting information

S1 Table Chemical secondary compounds concentration between plant tissue types. Correlations in chemical secondary compounds (mg/g of plant tissue) between tissue types of *Stryphnodendron adstringens*.
(PDF)
