# Supplementary material for: Trade-offs between growth, reproduction and defense in response to resource availability manipulations
Source: PLoS One. 2018 Aug 22;13(8):e0201873. doi: 10.1371/journal.pone.0201873 (PMC6104975; doi:10.1371/journal.pone.0201873)
Supplement: S1 Table — Correlations in chemical secondary compounds (mg/g of plant tissue) between tissue types of Stryphnodendron adstringens. (PDF) [file pone.0201873.s001.pdf]

Supporting information

S1 Table. Chemical secondary compounds concentration between plant tissue types. Correlations in chemical secondary compounds (mg/g of plant tissue) between tissue types of *Stryphnodendron adstringens*.

Chemical compound	Variables		*r*	*p*	*N*
Total phenols	Leaf total phenols	Fruit total phenols	0.09	0.604	38
	Leaf total phenols	Seed total phenols	-0.21	0.498	13
	Fruit total phenols	Seed total phenols	0.11	0.722	13
Hydrolyzable tannins	Leaf hydrolyzable tannins	Fruit hydrolyzable tannins	0.23	0.169	38
	Leaf hydrolyzable tannins	Seed hydrolyzable tannins	-0.09	0.733	13
	Fruit hydrolyzable tannins	Seed hydrolyzable tannins	-0.12	0.698	13
Condensed tannins	Leaf condensed tannins	Fruit condensed tannins	-0.16	0.326	38
	Leaf condensed tannins	Seed condensed tannins	0.21	0.497	13
	Fruit condensed tannins	Seed condensed tannins	0.11	0.722	13
